# Long‐term studies should provide structure for inclusive education and professional development

**DOI:** 10.1111/ele.14482

**Published:** 2024-12-31

**Authors:** Max F. Czapanskiy, Lina M. Arcila Hernández, Cara Munro, Isabella Garfield, Adrien Bastidas, Allison R. Payne, Kelli Ong, Natalie A. Storm, Taiki Adachi, Conner M. Hale, Astarte Brown, Patrick W. Robinson, Madeleine Stewart, Salma T. Abdel‐Raheem, Erika Zavaleta, Roxanne S. Beltran

**Affiliations:** ^1^ UC Santa Cruz, Institute for Marine Sciences Santa Cruz California USA; ^2^ UC Santa Cruz, Ecology and Evolutionary Biology Santa Cruz California USA; ^3^ National Institute of Polar Research Tachikawa Tokyo Japan

**Keywords:** broadening participation, diversity, equity, recruitment, retention, training, URM, workforce

## Abstract

Long‐term studies are critical for ecological understanding, but they are underutilized as inclusive opportunities for training ecologists. We use our perspective from the Año Nuevo elephant seal programme along with surveys from community members to propose that long‐term studies could be better leveraged to promote inclusive education and professional development in ecology. Drawing on our experiences as mentors and mentees, we demonstrate how long‐term studies can use their resources, including *rich data*, *robust logistics* and *extensive professional networks*, to improve recruitment and retention of diverse groups of trainees. However, practices such as unpaid labour and unclear expectations limit the utility of these resources for diversifying ecology. We discuss how we have structured our long‐term study to create more inclusive and equitable training opportunities. Acknowledging these transformations required substantial resources, we highlight funding sources and organizational partnerships that can promote investment in long‐term studies for broadening participation.

## Graphical abstract

Long‐term studies are valuable for the unique insights they provide into ecological systems, but their longevity also creates opportunities for creating inclusive training for future ecologists. In this viewpoint, we argue for an increased emphasis on equitable education among long‐term studies and highlight funding avenues supporting such work.

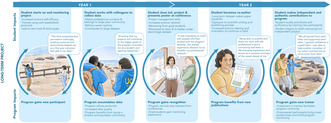

## PROBLEM STATEMENT

Long‐term ecological studies (exceeding 10 years) play a critical role in advancing scientific discovery (Clutton‐Brock & Sheldon, 2010; Hughes et al., 2017). These time‐ and labour‐intensive studies also employ many early career researchers. However, long‐term studies are typically designed around data collection goals, rather than to train a diverse workforce. Moreover, the prevalence of unpaid positions limits participation and retention (Fournier et al., 2019), contributing to the lack of diversity among ecologists (Chaudhury & Colla, 2021; Duc Bo Massey et al., 2021). As a result, we contend that long‐term studies are underutilized for inclusive training in ecology.

## UNDERUTILIZED ATTRIBUTES OF LONG‐TERM STUDIES

Equity‐minded, field‐based opportunities are powerful tools for diversity and inclusion in biology (Beltran et al., 2020; Zavaleta et al., 2020) because they support trainees as they develop their *science identity*, build confidence with skills (*self‐efficacy*) and generate a *sense of belonging* (Race et al., 2021; Shaulskiy et al., 2022; Shinbrot et al., 2022). Long‐term studies often possess characteristics like rich data, robust logistics and extensive professional networks that are valuable tools for supporting trainees. Although these characteristics are not unique to long‐term studies, we believe they are key components for long‐term studies to create inclusive educational opportunities.

**FIGURE 1:**
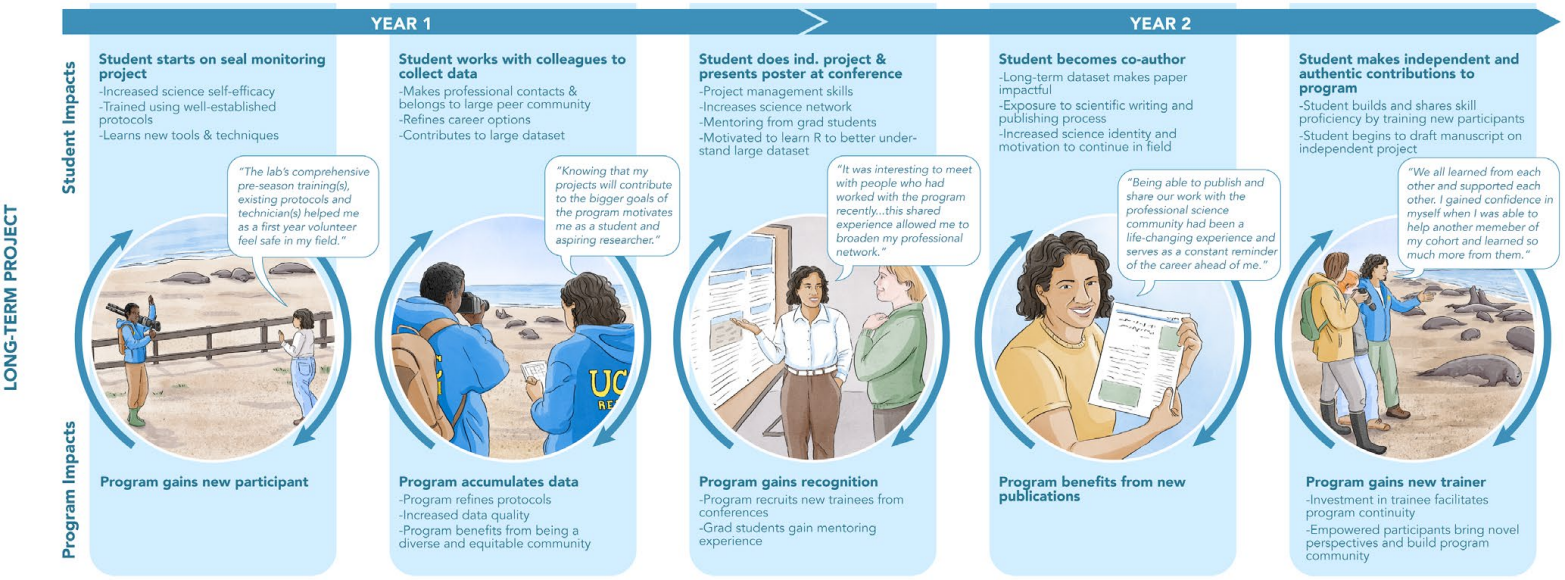
Inclusive training opportunities in long‐term studies create positive feedback loops for both students and programmes. Illustration by Alex Boersma.

The importance of rich data collected and curated by long‐term studies exceeds the data's initial scientific value (Figure 1). These data sets capture ecological variability across biological disciplines (e.g. demography and behaviour; Kratz et al., 2003; Lindenmayer et al., 2012). Because undergraduate research projects are often limited to months (such as a class or internship), the rich data from long‐term studies encourage students to investigate complex questions (Wishart et al., 2024). This is especially important for including students with accessibility accommodations and conflicting work or family commitments, who often face greater challenges to participating in fieldwork (Hall et al., 2004). Rich data from long‐term studies are a powerful motivator for students to analyse complex data sets, thereby developing their research skills and building *self‐efficacy*. Furthermore, when students contribute to long‐term study data collection, they join a scientific effort much larger than themselves, validating their *sense of belonging* and *science identity*.

Maintaining a long‐term study requires robust logistics such as vehicles, gear and training materials, which long‐term studies can use as tools to create inclusive entry points into ecology. All field ecology studies require logistics, but long‐term studies can refine theirs over time with the goal of broadening participation. Field gear costs (e.g. apparel and camping gear) and unspoken expectations for fieldwork are common barriers for new ecologists (Zavaleta et al., 2020). Long‐term studies can help address these obstacles by accumulating gear, such as binoculars and foul weather apparel, for loan to trainees. Transportation assistance to and from the study site, such as the volunteer “Farallon Patrol” that transports trainees to the Farallones long‐term study (McGlynn, 2012), can further reduce financial barriers. Robust onboarding and training are critical for setting transparent expectations and involving trainees equitably, rather than privileging those with the most prior experience (Emery et al., 2019; McGill et al., 2021; Figure 1). Although these logistics are possible in many field studies, the longevity of long‐term studies provides greater opportunities to refine their infrastructure and provide invaluable support for trainees.

Long‐term studies accumulate collaborators within and across disciplines, creating extensive professional networks that can connect students to multiple forms of mentorship. These professional networks frequently span career stages (e.g. principal Investigators and early career scientists) and career paths (e.g. academia and government agencies). When the networks also include community partners, their local knowledge and priorities can increase research salience and inclusion. While short‐term studies can also connect students to professional networks, the shared experiences between mentors and mentees in long‐term studies create rapport and strong community (Duffy & Gallagher, 2017; Wishart et al., 2024). As trainees gain experience in the programme, they benefit from the skills, data and mentorship provided by the network, potentially becoming mentors themselves (Figure 1). After trainees become programme alumni, they may benefit from other professional development opportunities (e.g. employment and education) circulated or facilitated by the network.

## EXPOSITION OF THE AUTHORS' VIEWS: A TRANSFORMATION IN PROGRESS

Many of us are part of the Año Nuevo (California, USA) long‐term study of northern elephant seals (*Mirounga angustirostris*). The Año Nuevo programme combines mark–recapture of individually marked seals (Le Boeuf et al., 2019) and satellite tracking of migrating animals (Robinson et al., 2012). Because of the programme's six‐decade history, it includes many principal investigators and an international network of collaborators in multiple disciplines. Therefore, in addition to facilitating impactful research, the Año Nuevo programme comprises an ideal opportunity to provide trainees with high‐quality learning opportunities through three avenues:

1. Our lab group trains approximately five undergraduate and approximately two graduate students per year as paid field assistants to collect data and undertake self‐directed research projects. After 5 weeks of regular trainings that bring faculty, staff, postdocs and students together, field assistants commit 5–10 h per week throughout one academic year to support the lab's long‐term monitoring and project‐specific goals.
2. Fourteen students per year enrol in a field‐based undergraduate course in which they participate in weekly elephant seal fieldwork with guidance from field assistants and undertake an independent research project using the elephant seal data set.
3. Partnership with existing diversity, equity and inclusion (DEI) programmes, such as the Doris Duke Conservation Scholars Program and the Center to Advance Mentored, Inquiry‐based Opportunities, allows us to offer paid summer internship programmes to students within and outside our university.

The value of this programme is substantial for both research and trainee success (Figure 1). To better understand the impact of the long‐term programme on trainees, we asked previous program participants to reflect on four properties of the elephant seal programme: rich data, robust logistics, professional networks and peer cohorts. We summarize these reflections and the impact of long‐term studies in Figure 1. For trainees, beginning the programme as a cohort supports social connections to peers and the rest of the programme. Hands‐on fieldwork facilitates a sense of community, science identity and project ownership that has been shown to attract and retain underrepresented minority students in STEM fields (Beltran et al., 2020).

We acknowledge the Año Nuevo programme has unique characteristics that may not reflect the resources and needs of other long‐term studies. Financially, we benefit from working in a well‐resourced institution in the global north and our study species is a research priority for funding agencies (e.g. the United States Office of Naval Research). Furthermore, the proximity of our study site to our institution (<35 km) reduces transportation costs relative to many other long‐term studies. However, we believe the feedback loop between creating equitable training opportunities and securing greater grant funding is a viable framework for other long‐term studies. In recent decades, the programme's budget has grown to include ca. $7200 for each of our 10 field assistants per year, in addition to growing numbers of graduate students and lab technicians (each ca. $70,000). These costs are covered by research grants that evolved to include more and more training emphasis—from student mentoring, to budgeting student salaries, to integrated research and training aims.

## ADDITIONAL PERSPECTIVES FROM THE RESEARCH COMMUNITY

To contextualize our experiences in the Año Nuevo programme with other long‐term studies, we created a survey for the other authors in this *Ecology Letters* special about their practices and goals for trainees. We received 27 responses from researchers around the world studying a broad range of organisms across ecosystems. In more than half of the responding programmes, undergraduate students collect, curate and analyse data, as well as develop research questions, present at conferences and contribute to writing scientific papers. Unlike our programme, which primarily recruits from one university, other long‐term studies often target a variety of institutions (79% of respondents). About half reported targeted recruitment of students from marginalized identities and a similar proportion paid students for their work. Students were slightly more likely to collaborate with a cohort of other students (3.8 in 1–5 Likert scale) and have peer cohort building activities (3.6 in 1–5 Likert scale). When asked to rank the helpful characteristics of long‐term studies to undergraduate students, top choices included “links between research, internship, classes”, “extensive professional networks”, “rich data” and “robust logistics”. The rank of “paid salaries” was split, with about a third of the programmes ranking it least helpful to students but the other third of programmes ranking it either as the first or second most helpful attribute of long‐term programmes. Based on comments related to funding, the low salary ranking might be associated with student funding opportunities available through university grants or independent fellowships. Overall, this suggests the most impactful transformation long‐term studies can make is to advocate for paying trainees in future grant proposals.

## TRANSFORMATIONS FOR INCLUSIVE AND FUNDABLE LONG‐TERM ECOLOGICAL STUDIES

**FIGURE 2:**
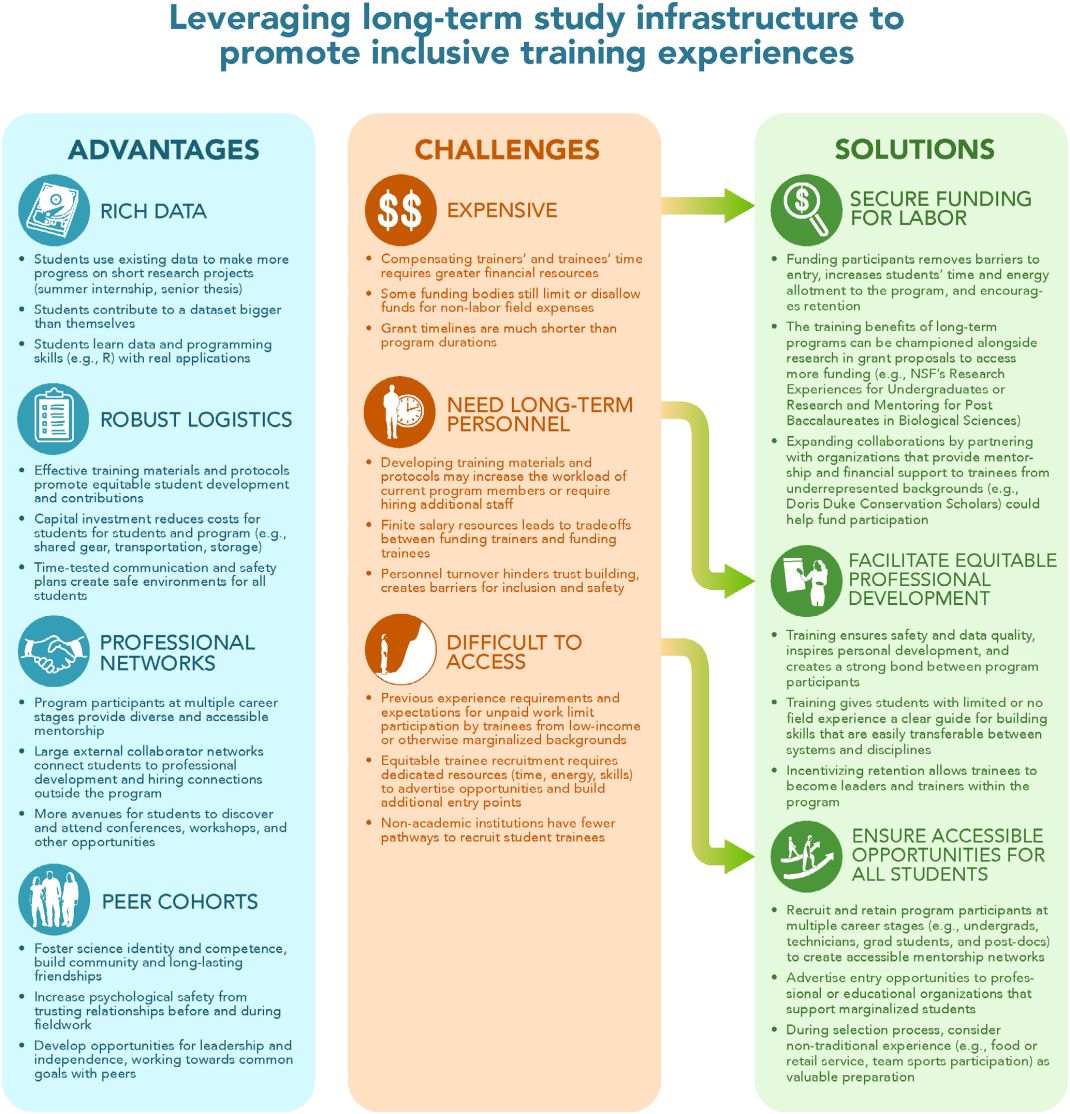
Creative solutions for securing funding, facilitating equity and ensuring access can help overcome the challenges of long‐term study continuation, so the advantages can be maximized. Illustration by Alex Boersma.

The infrastructure of long‐term ecological studies uniquely positions them to foster diversity, equity and inclusion in future generations of researchers. However, infrastructure alone is insufficient to realize this potential. Racism, sexism and other forms of oppression that exclude underrepresented minorities from field research and ecology exist in long‐term studies as well (Abdel‐Raheem et al., 2023; Jha, 2021; Viglione, 2020). The following transformations are tangible actions long‐term studies can use to affect material change in ecology (Figure 2). We have implemented some of these transformations in the Año Nuevo programme, but we are still evaluating their impact and their implementations may look different in other programmes. Although these recommendations are broadly applicable across field ecology studies, we believe the infrastructure of long‐term studies makes them particularly feasible and impactful.

Many new trainees are unfamiliar with the unspoken norms and expectations of fieldwork (i.e. the “hidden curriculum”; Giroux & Purpel, 1983; Jackson, 1990). Where to find an entry‐level opportunity, what to pack for the field, how to behave around wildlife: These critical steps to becoming an ecologist are rarely taught explicitly, which disproportionately excludes students with historically underrepresented identities (Arif et al., 2021). Dismantling the hidden curriculum in field research, by making opportunities and expectations explicit, can have immediate results for early career ecologists (Alwin et al., 2021; Zavaleta et al., 2020). Long‐term programmes can decrease these barriers for mentees by advertising opportunities at minority‐serving institutions, providing standardized protocols and setting expectations for field safety (Haacker, 2015).

Long‐term programmes can create peer cohorts among the trainees that persist across years. Cohorts in our case study consisted of 14 undergraduate students at a time; we recognize that, in most studies, cohort size might be smaller with only two to three trainees. Regardless of cohort size, creating structure for peer cohorts fosters both science identity and self‐efficacy, and developing those traits with peers builds community (Duffy & Gallagher, 2017; Wenger, 2000). Critically, community facilitates safety, as dangerous field conditions or interpersonal conflicts are exacerbated by isolation (Demery & Pipkin, 2021). The sense of community and safety from a peer cohort improves retention of underrepresented minorities (Dalbotten et al., 2014). Synchronizing onboarding, encouraging cross‐cohort mentoring and explicitly prioritizing camaraderie can build these peer cohorts. Partnering with cohort‐based DEI programmes can give long‐term studies access to expertise and resources needed to create equitable experiences for mentees.

Finally, one of the most impactful changes available to long‐term programmes is paying for all labour, including undergraduates. Volunteer positions in ecology exclude many participants and devalue our science (Fournier & Bond, 2015). The burdens of unpaid work are often more pronounced for women of colour from low socioeconomic backgrounds (Fournier et al., 2019). Students and recent graduates with paid STEM positions are more likely to persist in their selected fields and find meaningful employment afterwards (Bailey et al., 2022; Fournier et al., 2019). The path forward for programme leaders is to budget for trainees alongside other necessary expenses. Many funding agencies include DEI and personnel training as stated priorities. For example, National Science Foundation funding for broadening participation has tripled over the last decade (National Science Foundation, 2023) and “training of highly qualified personnel” accounts for one‐third of the evaluation for Natural Sciences and Engineering Research Council of Canada (NSERC) grant proposals.

## CONCLUSION

Long‐term studies play an important role in ecology, but they could make a greater impact creating inclusive training opportunities. Time and funding constraints will forever necessitate a difficult trade‐off between conducting research and training researchers. We hope that our proposed transformations provide actionable solutions balancing long‐term research and inclusion (Figure 2). As funding agencies increasingly prioritize broadening participation, long‐term studies should emphasize the benefits of budgeted infrastructure, including experienced personnel, for training and equity. With these transformations, long‐term studies will have greater impacts on ecology by providing equitable training opportunities for future generations of scientists.

## AUTHOR CONTRIBUTIONS

MFC, LMAH and RSB conceived the viewpoint and led writing of the manuscript; CM, IG, AB, ARP, KO, NAS, CMH, AB, MS and STAR contributed text to the draft; RSB and EZ provided supervision and feedback; LMAH conducted the survey; all authors contributed substantially to revisions.

### PEER REVIEW

The peer review history for this article is available at https://www.webofscience.com/api/gateway/wos/peer‐review/10.1111/ele.14482.

## Data Availability

Aggregated survey response data are available on Dryad (doi:10.5061/dryad.7sqv9s51h).
